# The ε-Isozyme of Protein Kinase C (PKCε) Is Impaired in ALS Motor Cortex and Its Pulse Activation by Bryostatin-1 Produces Long Term Survival in Degenerating SOD1-G93A Motor Neuron-like Cells

**DOI:** 10.3390/ijms241612825

**Published:** 2023-08-15

**Authors:** Valentina La Cognata, Agata Grazia D’Amico, Grazia Maugeri, Giovanna Morello, Maria Guarnaccia, Benedetta Magrì, Eleonora Aronica, Daniel L. Alkon, Velia D’Agata, Sebastiano Cavallaro

**Affiliations:** 1 Institute for Biomedical Research and Innovation, National Research Council, 95126 Catania, Italy; 2 Section of Human Anatomy and Histology, Department of Biomedical and Biotechnological Sciences, University of Catania, 95123 Catania, Italy; 3 Department of (Neuro) Pathology, Amsterdam UMC, University of Amsterdam, Amsterdam Neuroscience, Meibergdreef 9, 1105 Amsterdam, The Netherlands; 4 Synaptogenix, Inc., New York, NY 10036, USA

**Keywords:** PKCε, *PRKCE*, amyotrophic lateral sclerosis, neurodegeneration, Bryostatin-1

## Abstract

Amyotrophic lateral sclerosis (ALS) is a rapidly progressive and ultimately fatal neurodegenerative disease, characterized by a progressive depletion of upper and lower motor neurons (MNs) in the brain and spinal cord. The aberrant regulation of several PKC-mediated signal transduction pathways in ALS has been characterized so far, describing either impaired expression or altered activity of single PKC isozymes (α, β, ζ and δ). Here, we detailed the distribution and cellular localization of the ε-isozyme of protein kinase C (PKCε) in human postmortem motor cortex specimens and reported a significant decrease in both PKCε mRNA (*PRKCE*) and protein immunoreactivity in a subset of sporadic ALS patients. We furthermore investigated the steady-state levels of both pan and phosphorylated PKCε in doxycycline-activated NSC-34 cell lines carrying the human wild-type (WT) or mutant G93A SOD1 and the biological long-term effect of its transient agonism by Bryostatin-1. The G93A-SOD1 cells showed a significant reduction of the phosphoPKCε/panPKCε ratio compared to the WT. Moreover, a brief pulse activation of PKCε by Bryostatin-1 produced long-term survival in activated G93A-SOD1 degenerating cells in two different cell death paradigms (serum starvation and chemokines-induced toxicity). Altogether, the data support the implication of PKCε in ALS pathophysiology and suggests its pharmacological modulation as a potential neuroprotective strategy, at least in a subgroup of sporadic ALS patients.

## 1. Introduction

Amyotrophic lateral sclerosis (ALS) is a fatal adult-onset neurodegenerative disorder characterized by the progressive degeneration of upper and lower motor neurons (MNs) in the cortex, brainstem and spinal cord. Motor neuron deterioration results in muscle weakness and, ultimately, in death due to respiratory failure, typically within 3–5 years after diagnosis [1,2].

The majority of cases (90%) are sporadic (SALS) without a family history, while the remaining 10% of ALS patients are inherited (familial ALS or FALS) [1,3,4]. Approximately 12% of familial cases and 2% of sporadic ALS cases are caused by mutations in the Cu/Zn superoxide dismutase 1 (*SOD1*) gene, one of the first discovered ALS genes [5,6,7,8]. The clinical presentation of SALS and FALS are similar, and treatment options remain primarily supportive so far. Indeed, the two current FDA-approved drugs, i.e., the anti-excitotoxic Riluzole (Rilutek) and the antioxidant Edaravone, are able to extend the lifespan of patients by a few months and counteract disease progression without a real resolutive outcome [9,10].

The pathogenic process underlying ALS neurodegeneration is multifactorial and still not fully determined, although dysfunctions in several cellular and molecular processes have been reported so far, including impaired protein homeostasis, mitochondrial alterations, aberrant RNA metabolism, neuroinflammation, excitotoxicity and oxidative stress [11]. In the last few years, our research group and others have demonstrated that sporadic ALS is a phenotypically and genetically heterogenous disease, and SALS patients may be taxonomized into distinct molecular subtypes based on postmortem motor cortex transcriptomic signatures [11,12,13,14,15,16]. This evidence emphasized the idea that molecular-based studies aimed at uncovering the disease etiopathogenesis, as well as at characterizing biomarkers or effective treatments, require updating and necessitate accurate stratified case monitoring [11].

Multiple studies have implicated deregulation in ALS of the protein kinase C (PKC)-mediated signal transduction mechanisms, through changes in either the expression or activity state of several members of the PKC superfamily [17,18,19,20,21,22]. This latter consists of 10 related serine/threonine protein kinases (isozymes) that can be grouped into three subclasses, according to structural motifs and activation requirements: (i) classical (also termed conventional) cPKCs (α, β and γ) require both diacyl glycerol (DAG) and a calcium ion for activation, (ii) novel nPKCs (δ, ε, η and θ) require DAG but not by calcium [23], and (iii) atypical aPKCs (ζ and τ/γ) are insensitive to calcium and DAG but are activated by other lipids or by phosphorylation [23,24].

The novel ε isoform (PKCε) is a finely regulated enzyme known for its important roles in the nervous [25,26], cardiac [27] and immune systems [28]. Currently, it represents an attractive target for the treatment of several conditions, such as inflammation, ischemia, addiction, pain, anxiety and cancer [24], and has recently gained attention in Alzheimer’s disease (AD) for its role in both memory formation and regulation of β-amyloid misfolded proteins [29,30]. The PKCε enzyme shares many structural features with the other members of the PKC family, including the DAG (C1) and the C2-like phospholipid-binding domains, the pseudo-substrate (PS) site, the catalytic terminal C3 and C4 domains containing the ATP binding site, the substrate recognition site and the kinase domain [24]. Like the other PKC isozymes, PKCε must be primed through phosphorylation to display full enzymatic activity and respond to allosteric regulators. Phosphorylation can occur at three conserved sites in the catalytic domain: the activation loop (Thr-566), the Thr-Pro turn motif (Thr-710) and the hydrophobic Phe-Ser-Tyr motif (Ser-729) [24]. Following activation, PKCε translocases into specific subcellular compartments (e.g., perinuclear/Golgi site) and changes the substrate kinetics [31].

One of the most potent PKCε activators is the marine natural product Bryostatin-1, a macrocyclic lactone originally isolated from *Bugula neritina*. This molecule has long been investigated in neuroscience for its interesting ameliorative effects on neuronal structure and function in in vitro studies, as well as for the pro-cognitive and antidepressant outcomes in vivo in animal models, thus entering into human clinical trials for treating AD [32,33]. Bryostatin-1 produces a time-dependent biphasic effect on PKCε levels: firstly, it binds and activates PKCε, promoting its translocation from cytosol to membrane fractions [34]; then, PKCε is proteolytically degraded during the so-called downregulation step and, lastly, undergoes a phase of de novo protein synthesis which restores PKCε normal levels and induces the production of additional trophic factors (e.g., BDNF) [32,34].

The aberrant regulation of α, β, ζ and δ PKC isozymes in ALS has been previously described [17,18,19,20,21,22,35,36], but nothing is known about the contribution of the ε isoform in the ALS pathophysiology. In the present work, we investigated the PKCε mRNA (*PRKCE*) expression level and the PKCε protein cellular expression and localization in human postmortem motor cortex specimens from control and ALS patients’ subtypes. Furthermore, we evaluated the steady-state levels of pan and phosphorylated PKCε in murine NSC-34 motor neuron-like cells expressing human wild-type (WT) or mutant G93A-SOD1 [37] and inspected the biological long-term effect of PKCε activation by Bryostatin-1.

## 2. Results

### 2.1. PKCε Is Expressed by Different Cell Types in Human Primary Motor Cortex

**Figure 1:**
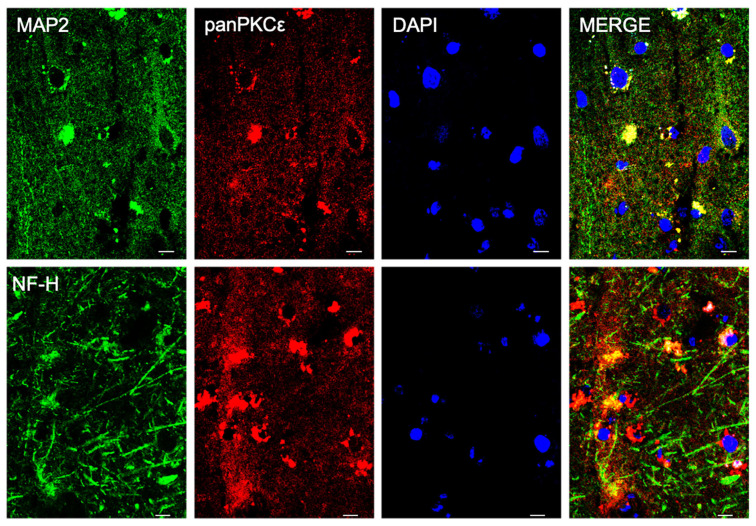
PKCε is expressed by cortical neurons in human primary motor cortex. Representative photomicrographs show panPKCε expressed in cortical neuronal cells (MAP2^+^ or NF-H^+^) examined under a Nikon A1 confocal inverted microscope equipped with a Plan Apochromat lambda 60×/1.4 oil immersion lens (Nikon, Tokyo, Japan). Scale bar 10 μm.

**Figure 2:**
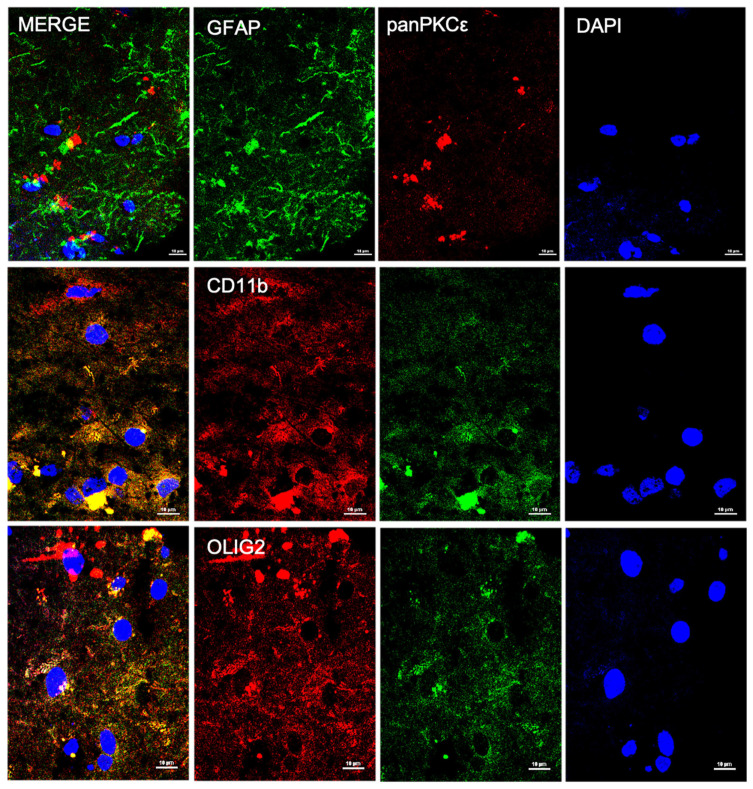
PKCε is expressed by non-neuronal cells in human primary motor cortex. Representative photomicrographs show panPKCε expressed in microglial cells (CD11b^+^) and oligodendrocytes (OLIG2^+^) but barely in astrocytes (GFAP^+^). Slides were examined under a Nikon A1 confocal inverted microscope equipped with a Plan Apochromat lambda 60×/1.4 oil immersion lens (Nikon, Tokyo, Japan). Scale bar 10 μm.

In order to understand the biological role of PKCε in the pathophysiology of the human motor cortex, we first investigated its cellular distribution in postmortem cortical specimens from control patients by fluorescence immunohistochemistry. Double labeling with fluorescent antibodies revealed a widely panPKCε immunoreactivity in the cortical neurons (MAP2^+^ or NF-H^+)^, microglial cells (CD11b^+^) and oligodendrocytes (OLIG2^+^), but barely in the astrocytes (GFAP^+^) (Figure 1 and Figure 2).

### 2.2. PRKCE mRNA Expression Level Is Reduced in Motor Cortex in a Subset of ALS Patients

To characterize the biological significance of PKCε in ALS, we first compared the expression level of PKCε encoding-gene (*PRKCE*) in control and ALS motor cortex subgroups from two independent RNA gene-expression studies.

**Figure 3:**
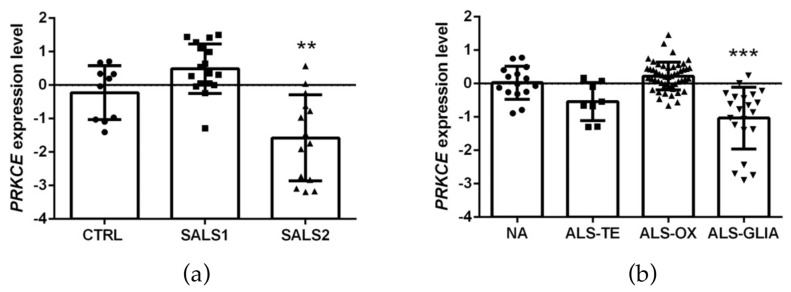
PKCε mRNA (*PRKCE*) is downregulated in motor cortex in a subset of sporadic ALS patients. (**a**). Transcriptomic data extrapolated from E-MTAB-2325 dataset showing a statistically significant downregulation of *PRKCE* mRNA expression level in motor cortex in a subset of sporadic ALS patients (SALS2) compared to CTRL (control = 10, SALS1 = 18, SALS2 = 13 patients, respectively). (**b**). Transcriptomic data derived from GSE124439 dataset showing a statistically significant downregulation of *PRKCE* mRNA expression level in motor cortex in a subset of ALS patients (ALS-Glia) compared to non-neurological controls (NA) (NA = 15, ALS-TE = 8, ALS-OX = 51, ALS-Glia = 21 patients, respectively). Data were extrapolated from ArrayExpress (http://www.ebi.ac.uk/arrayexpress/ accessed on 1 June 2021) and Gene Expression Omnibus (https://www.ncbi.nlm.nih.gov/geo/ accessed on 1 January 2023), respectively, and analyzed as described in the Materials and Methods section. Tukey–Kramer post hoc test: ** *p* < 0.01 vs. CTRL, *** *p* < 0.001 vs. NA. Circles, squares or triangles indicate the *PRKCE* expression level of single patients for each experimental group from the two datasets.

The first analysis relied on the E-MTAB-2325 transcriptomic dataset, which collected the whole-genome microarray RNA profiles of the motor cortex from 31 sporadic ALS samples and 10 controls. The previous examination of these RNA profiles had revealed a clear transcriptional-based clustering of subjects into three distinct groups: control (*n* = 10), SALS1 (*n* = 18) and SALS2 (*n* = 13) subtypes, each associated with different molecular features and potential drug targets [14,16,38]. Among the multiple differentially expressed genes, *PRKCE* emerged as significantly decreased in SALS2 (not in SALS1) patients compared to the controls (Figure 3a).

To corroborate this observation, we further explored the cortical *PRKCE* mRNA level in a second bulk transcriptome study (i.e., the GSE124439 dataset), which profiled by RNA-sequencing 80 ALS and 15 non-neurological control (NA) motor cortex areas (both medial and lateral) [15], and stratified the ALS patients into three distinct molecular subtypes: (i) ALS-TE, marked by retrotransposon re-activation as a dominant feature (*n* = 8); (ii) ALS-OX, showing evidence of oxidative and proteotoxic stress (*n* = 51); (iii) ALS-Glia, with strong signatures of glial activation and inflammation (*n* = 21) [15]. Interestingly, a significant downregulation of *PRKCE* mRNA was observed only in the ALS-Glia patients (Figure 3b).

### 2.3. PKCε Immunoreactivity Is Decreased in Both ALS Postmortem Primary Motor Cortex and SOD1-G93A NSC-34 Cells

**Figure 4:**
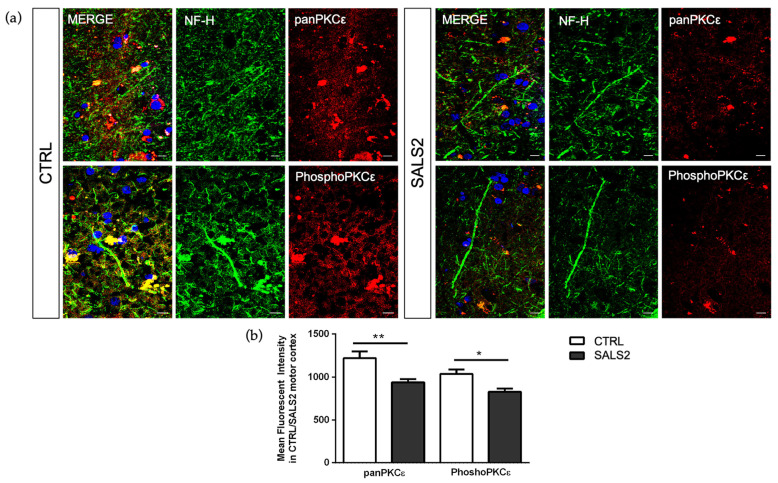
The panPKCε and phosphoPKCε levels are reduced in motor cortex area of SALS2 patients. (**a**). Representative images showing panPKCε and phosphoPKCε immunoreactivity in motor cortex area (characterized by NF-H positive staining) of control and SALS2 patients. (**b**). Fluorescence mean intensity, quantified by examining samples under a Nikon A1 confocal inverted microscope equipped with a Plan Apochromat lambda 60×/1.4 oil immersion lens (Nikon, Tokyo, Japan). The mean intensity of TRITC channel was extrapolated from multiple regions of interest (ROI) and normalized to the background by using the NIS-Elements AR (Advanced Research) software (version 4.60). Scale bar 10 μm. Tukey–Kramer post hoc test: ** *p* < 0.01 SALS2 vs CTRL for panPKCε, * *p* < 0.05 SALS2 vs. CTRL for phosphoPKCε.

To characterize the global protein expression and phosphorylation state of PKCε in the human control and SALS2 motor cortex samples, we performed fluorescent immunohistochemistry studies. Staining with both anti-panPKCε and anti-phosho-S729-PKCε antibodies revealed an overall decreased immunoreactivity for both antibodies in the motor cortex (NF-H^+^ area) of SALS2 patients compared to controls (Figure 4).

**Figure 5:**
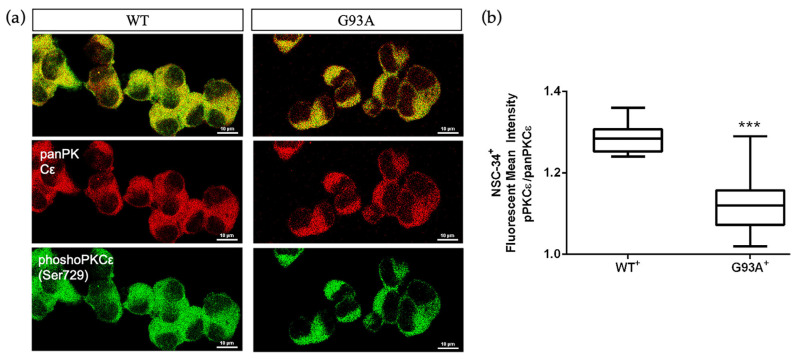
Immunoreactivity ratio of phosphoPKCε/panPKCε is reduced in SOD1-G93A NSC-34 cells. (**a**). Representative images showing panPKCε and phosphoPKCε immunoreactivity in WT and SOD1-G93A NSC-34 cells. (**b**). Fluorescence mean intensity, quantified by examining samples under a Nikon A1 confocal inverted microscope equipped with a Plan Apochromat lambda 60×/1.4 oil immersion lens (Nikon, Tokyo, Japan). The mean intensity of each channel was extrapolated from multiple regions of interest (ROI) and normalized to the background by using the NIS-Elements AR (Advanced Research) software (version 4.60). Scale bar 10 μm. Tukey–Kramer post hoc test: *** *p* < 0.001 vs. WT.

Considering that the SALS2 subcluster was the only one showing significant deregulation in SOD1 expression level [16,38], we decided to inspect PKCε expression in vitro in the widely used murine cellular humanized ALS model, i.e., NSC-34 over-expressing WT or mutated human SOD1-G93A under doxycycline activation, as previously reported [39]. Consistent with the human-derived motor cortex data, we detected a downregulation of the panPKCε and phosphoPKCε immunoreactivity in G93A NSC-34 cells compared to WT (Figure 5) and used this model for the following studies.

### 2.4. A Pulse Activation by Bryostatin-1 Promotes Long-Term Cell Survival in Degenerating SOD1-G93A NSC-34 Cells and Changes the phosphoPKCε/panPKCε Ratio

**Figure 6:**
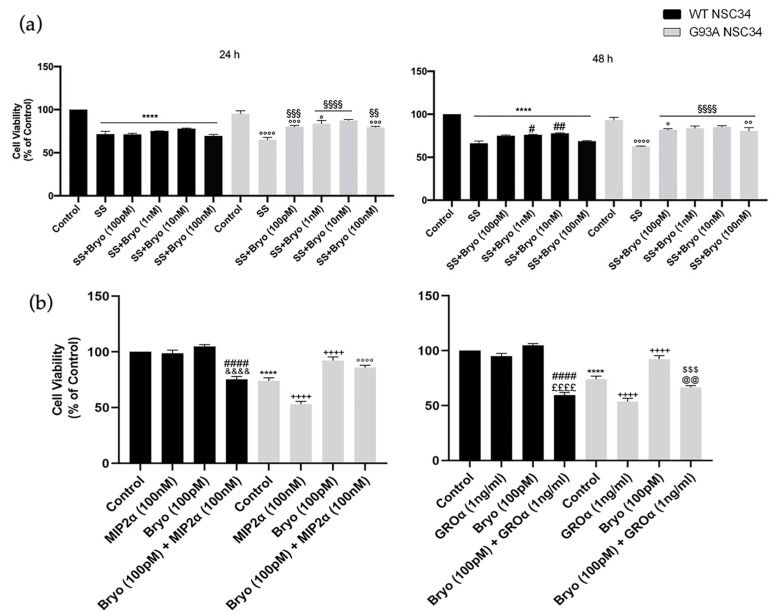
A pulse activation of PKCε by Bryostatin-1 produces long-term survival in degenerating mutant SOD1-G93A cells. (**a**). Cell viability of WT and SOD1-G93A NSC-34 cultured in normal growth medium (Control), serum starvation (ss) and exposed at different concentrations of Bryostatin-1 for 10 min after 24 and 48 h. Normal growth medium-cultured cells were used as controls. Results are representative of at least three independent experiments and values are expressed as a percentage of control (**** vs. Control WT, ^°^ or ^°°^ or ^°°°^ or ^°°°°^ vs. Control G93A, ^#^ or ^##^ vs. SS WT, ^§§^ or ^§§§^ or ^§§§§^ vs. SS G93A as determined by one-way ANOVA followed by Tukey–Kramer post hoc test). (**b**). Cell viability of WT and SOD1-G93A NSC-34 cultured for 48 h in normal growth medium (Control), in combination with toxic chemokines (GROα and MIP2α) and exposed to Bryostatin-1 (100 pMol) for 10 min. Normal growth medium-cultured cells were used as controls. Results are representative of at least three independent experiments and values are expressed as a percentage of control (**** *p* < 0.0001 vs. control WT, ^++++^ *p* < 0.0001 vs. control G93A, ^####^ *p* < 0.0001 vs. Bryo WT, ^$$$^ *p* < 0.001 vs. Bryo G93A, ^°°°°^ *p* < 0.0001 vs. MIP2α G93A, ^&&&&^ *p* < 0.0001 vs. MIP2α WT, ^££££^ *p* < 0.0001 vs. GROα WT, ^@@^ *p* < 0.0001 vs. GROα G93A as determined by one-way ANOVA followed by Tukey–Kramer post hoc test).

Based on the observed PKCε downregulation in the ALS condition, we wondered about the downstream pharmacological effects of PKCε agonism in vitro and examined the biological outcomes of PKCε pulse activation by Bryostatin-1 in WT and G93A NSC-34 cells in two different paradigms of cell death models. Induction to apoptosis in both WT and G93A doxy-activated cells was prompted by: (i) serum starvation at either 24 or 48 h, or (ii) co-incubation with toxic chemokines (i.e., MIP2α and GROα) for 48 h. This second apoptosis model derives from previous observations conducted in our laboratories [40], which highlighted the vulnerability of G93A cells to MIP2α and GROα ligand treatment. Indeed, in the chemokines-induced cell death paradigm, the G93A NSC-34 cells displayed more sensitivity to apoptosis compared to the WT NSC-34, showing a peculiar significant reduction of cell viability in the presence of GROα and MIP2α (Figure 6b).

The cells were then exposed to a pulse treatment (10 min) with Bryostatin-1. We used increasing concentrations of Bryostatin-1 (100 pM, 1 nM, 10 nM and 100 nM) in the serum deprivation condition (Figure 6a) and a single dose (100 pM) in the chemokines-induced toxicity paradigm (Figure 6b). In both apoptotic paradigms, the PKCε pulse activation by Bryostatin-1 determined a significant increase in cellular viability in degenerating G93A NSC-34 cells compared to the untreated controls (Figure 6a).

**Figure 7:**
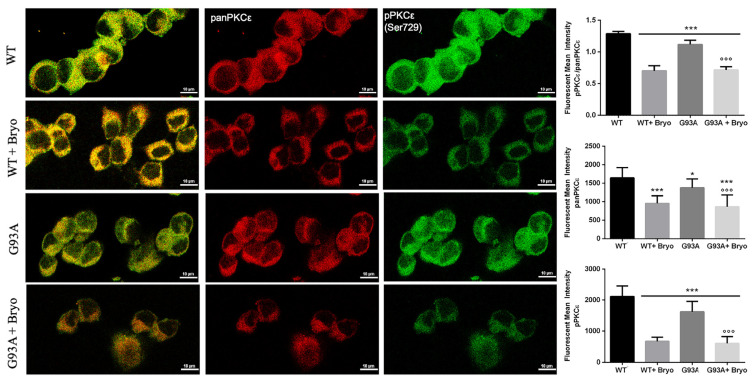
A transient treatment with Bryostatin-1 induces reduction of PKCε expression level in both WT and SOD1-G93A cells. Representative images showing panPKCε and phosphoPKCε immunoreactivity in WT and SOD1-G93A NSC-34 cells after 48 h from the 10 min pulse activation by Bryostatin-1. Fluorescence was quantified by examining samples under a Nikon A1 confocal inverted microscope equipped with a Plan Apochromat lambda 60×/1.4 oil immersion lens (Nikon, Tokyo, Japan). The mean intensity of each channel was extrapolated from multiple regions of interest (ROI) and normalized to the background by using the NIS-Elements AR (Advanced Research) software (version 4.60). Scale bar 10 μm. Tukey–Kramer post hoc test: *** *p* < 0.001 or * *p* < 0.05 vs. WT, °°° *p* < 0.001 vs. G93A.

Previous data on Bryostatin-1 showed it produces a time-dependent biphasic effect on PKCε levels since it immediately binds PKCε promoting its self-phosphorylation and translocation from cytosol to membrane fractions [34,41], and then the enzyme undergoes a downregulation phase for several hours, followed by de novo synthesis. We therefore measured PKCε immunoreactivity after 48 h from Bryostatin-1 pulse activation and, concordantly with previous observations, detected late decreased levels of the phosphoPKCε/panPKCε ratio in both the WT and G93A NSC-34 cells (Figure 7).

## 3. Discussion

The mechanisms underlying motor neuron cell death and axonal degeneration in ALS still remain elusive, partly due to our incomplete knowledge of the biological mechanisms controlling neuronal degeneration. In this study, we focused our attention on the ε-isozyme of PKC (PKCε), a versatile enzyme regulating a number of cellular processes including proliferation, differentiation, chemotaxis, neurogenesis of cortical area, outgrowth of neurites, memory, synaptic growth and synaptogenesis, and mitochondria-mediated regulation of free radical production and apoptosis [17,42,43,44,45,46].

PKCε is widely expressed throughout the body, predominantly in brain regions [25,36,47] such as the hippocampus, Calleja’s islands, olfactory tubercle, cerebral cortex, septal nuclei, nucleus accumbens, frontal cortex, striatum and caudate putamen [45]. In the present study, we detailed PKCε distribution in the postmortem human primary motor cortex, describing its expression in cortical neurons (MAP2^+^ or NF-H^+^), microglial cells (CD11b^+^) and oligodendrocytes (OLIG2^+^), but barely in astrocytes (GFAP^+^).

Despite a number of former studies highlighted a significant deregulation of other PKC- isozymes (α, β, ζ and δ) in the motor neurons of ALS patients and in SOD1-G93A murine models [17,18,19,35,36], no previous studies have investigated the role of the ε-isozyme in ALS pathophysiology. Here, we observed that PKCε mRNA expression level does not show differences when ALS is considered as a single entity, while it displays a significant downregulation in particular molecular subtypes of sporadic ALS patients obtained by bulk transcriptomic-based profiling (SALS2 from Aronica et al. [16] and ALS-Glia subset from Tam et al. [15]). Then, focusing the immunofluorescence analysis on postmortem SALS2 primary motor cortex areas, we disclosed a concordant significant downregulation of both panPKCε and phospho-Ser729-PKCε expression compared to the controls. Such PKCε downregulation may be the result of the selective motor neuronal depletion in terminal ALS patients, which are usually characterized by extensive astrocytosis.

As previously described, the SALS2 subcluster was the only one showing significant deregulation of the *SOD1* expression level [16,38]. Therefore, we decided to inspect PKCε-mediated biological effects in an ALS in vitro model characterized by overexpression of WT and mutant SOD1, i.e., NSC-34 carrying WT or mutated human SOD1 (G93A). Consistent with the human-derived motor cortex data, we detected a downregulation of phosphoPKCε/panPKCε ratio immunoreactivity in G93A NSC-34 cells compared to WT.

Given the decreased PKCε expression and its impaired phosphorylation state in ALS, we investigated the long-term biological effect of Bryostatin-1, a macrolide lactone and potent agonist of PKCε [32] in both WT and G93A NSC-34 motor neuron-like cells [37], triggered to death by two different apoptotic ways (growth factor starvation and chemokines-induced toxicity). The in vitro assays revealed that a PKCε pulse activation treatment (10 min) by Bryostatin-1 plays a long-term neuroprotective action in degenerating cells, especially in the G93A background. This finding is in agreement with previous studies showing that Bryostatin-1 increases cortical synaptogenesis and is useful in enhancing learning and memory in preclinical models of AD [32,33,48]. Moreover, the transient brief activation was sufficient to prompt a shift down of the phosphoPKCε/panPKCε ratio level in both WT and mutant G93A NSC-34 cells, a finding that could represent the well-documented downregulation phenomenon resulting from PKCε C1 domain activation in neurons [32,49,50,51].

Of course, the study has several limitations, such as the unknown precise time course of PKCε turnover, and the used murine cell-based model, which does not exhaustively recapitulate the complex ALS portrait. Nonetheless, these findings, along with the well-characterized multiplicity of PKCε functions and variation in cellular and tissue distribution, could raise some interesting considerations about the contribution of the ε isozyme kinase in the pathogenesis of ALS [21]. Indeed, a kinase alteration could impact the production of trophic factors (e.g., BDNF) for neuronal survival, cell cycle checkpoints regulating neuronal death and survival, axonal transport and the stimulation of excitatory amino acid receptors and Ca^2+^ channels [22]. Moreover, in other neurodegenerative diseases, Bryostatin-1 proved to revert synaptic loss and restore cognitive functions [41,52]. In Alzheimer’s models and patients, for example, it is able to increase synaptogenesis through the increase in BDNF, and, therefore, it is emerging as a potential neuroprotective treatment [41,52].

Although the mechanisms described in this work are still preliminary, and the number of analyzed patients is few, the results encourage additional preclinical and clinical investigations to guide new directions in the knowledge of ALS pathophysiology. Moreover, since deregulated expression of *SOD1* was exclusively found in SALS2 but not in SALS1 patients [16,38], and the sporadic ALS-Glia human subset shares some transcriptional signatures with murine SOD1-G93A spinal microglia [15,53], SOD1-G93A NSC-34 may represent a suitable preclinical model to investigate a distinct subset of ALS human pathology.

## 4. Materials and Methods

### 4.1. Transcriptomic Profiling

For this study, we referred to a previously described bulk transcriptome dataset [16,54] available at ArrayExpress (http://www.ebi.ac.uk/arrayexpress/ accessed on 1 June 2021) with the accession number E-MTAB-2325 (https://www.ebi.ac.uk/biostudies/arrayexpress/studies/E-MTAB-2325/ accessed on 1 June 2021) The dataset consists of the expression profiles of motor cortexes from SALS (*n* = 31) and control (*n* = 10) subjects produced with 4 × 44 K Whole Human Genome Oligo expression microarrays (Agilent Technologies, Santa Clara, CA, USA). A detailed description of the subject characteristics (origin, source code, age, gender, race, disease state, survival time from diagnosis date and postmortem interval) and experimental procedures have been previously reported [16,54,55]. Raw intensity signals from motor cortex sample hybridization were thresholded to 1, log2-transformed, normalized and baselined to the median of all the samples by using GeneSpring GX (Agilent Technologies, Santa Clara, CA, USA). Values from probes targeting *PRKCE* were extrapolated for the following analysis.

To further investigate the *PRKCE* mRNA levels in the ALS motor cortex, we used a second independent transcriptome study (GSE124439 dataset), which profiled, by RNA sequencing, a number of frontal and motor cortex specimens from a large cohort of ALS (*n* = 148) and non-neurological (NA) subjects (*n* = 28) [15]. The data from this dataset were downloaded from the Gene Expression Omnibus (https://www.ncbi.nlm.nih.gov/geo/ accessed on 1 January 2023), imported on GeneSpring GX (Agilent Technologies), thresholded to 1 and baselined to the median of all the samples. Signals from ALS (*n* = 80) and control (*n* = 15) primary motor cortex (both medial and lateral) samples were used for further analysis.

### 4.2. Fluorescent Immunohistochemistry

Postmortem frozen sections (10 μm) of motor cortex samples from control and ALS patients were collected and processed in order to perform immunofluorescence analyses, as described elsewhere [12,16,56]. We used the following primary antibodies: anti-PKCε (PA5-102580, Thermo Fisher Scientific, Waltham, MA, USA, 1:200 and sc-1681, Santa Cruz Biotechnology, Inc. Dallas, TX, USA, 1:250), anti-phosho-S729-PKCε (#44-977G, Thermo Fisher Scientific, Waltham, MA, USA, 1:250), anti-MAP2 (M13-13-1500, Thermo Fisher Scientific, Waltham, MA, USA, 1:300), anti-NF-H (ab187374, Abcam, Cambridge, UK, 1:200), anti-CD11b (ab133357, Abcam, Cambridge, UK, 1:500) and anti-GFAP (MAB360, Merck Millipore, Burlington, MA, USA, 1:500). TRITC- and FITC-conjugated secondary antibodies (Goat anti-rabbit 111-025-003 and Goat anti-mouse 115-095-003, Jackson Laboratories Inc., Baltimore, PA, USA) were used for 1 h at room temperature in the dark. The slides were washed 3 times in PBS after every step, mounted with glycerol mounting medium containing DAPI and analyzed with a Nikon A1 confocal inverted microscope equipped with a Plan Apochromat lambda 60×/1.4 oil immersion lens (Nikon, Tokyo, Japan). Fluorescence was quantified by analyzing the mean intensity of each channel from multiple regions of interest (ROI), normalized to the background by using the NIS-Elements AR (Advanced Research, Nikon, Tokyo, Japan) software (version 4.60).

### 4.3. Cell Culture

A mouse motor neuron-like hybrid NSC-34 cell line (kindly provided by Dr. Cinzia Volontè from the National Research Council, Institute for Systems Analysis and Computer Science “Antonio Ruberti”) [57] was stably transfected with the pTet-ON plasmid (Clontech, Palo Alto, CA, USA) coding for the reverse tetracycline-controlled transactivator, used to construct inducible cell lines expressing the cDNAs encoding human wild-type-SOD1 (WT) or human SOD1 mutant G93A (SOD1-G93A), as previously described [39,58], and listed hereafter as WT and G93A NSC-34 cells. The treatment with doxycycline (2 µg/mL) for 24 h was used to induce WT and mutant G93A SOD1 expression.

### 4.4. Immuno-Cytofluorescence

NSC-34 cells expressing human WT or SOD1-G93A were cultured on glass cover slips, fixed in 4% paraformaldehyde and processed in order to perform an immunofluorescence assay [12,59]. The samples were probed with specific primary antibodies: anti-PKCε (sc-1681, Santa Cruz Biotechnology, Inc. Dallas, TX, USA, 1:200), anti-phosho-S729-PKCε (#44-977G, Thermo Fisher Scientific, Waltham, MA, USA, 1:200); Alexa Fluor 488 Goat anti-rabbit and Alexa Fluor 594 Goat anti-mouse were used as secondary antibodies (Jackson Immuno-research). The analyses were performed by using confocal microscopy, as reported elsewhere [60]. The fluorescence was quantified by extrapolating the mean intensity of each channel from multiple regions of interest (ROI) and normalized to the background [12] by using the NIS-Elements AR (Advanced Research) software.

### 4.5. Cellular Viability Assay

Cell viability was assessed using the colorimetric reagent-based MTT cell proliferation kit I, based on the 3-[4,5-dimethylthiazol-2-yl]-2,5-diphenyltetrazolium bromide (Roche Diagnostics, Germany) salt, as previously described [58,61]. Briefly, after 24 hours from the doxycycline (2 µg/mL) induction, the cells were prompted to apoptosis by serum starvation or chemokines-induced toxicity (GROα, 1 ng/mL and MIP2α, 100 nM) (SRP4210 and SRP4251, Sigma-Aldrich, Munich, Germany). The cells were incubated for 10 min (pulse treatment) with Bryostatin-1 at different concentrations (100 pM, 1 nM, 10 nM and 100 nM, Calbiochem, Merck Millipore, Burlington, Massachusetts) and allowed to grow for 24 or 48 h. Subsequently, 0.5 mg/mL of MTT was added to each well and incubated for 4 h at 37 °C. The reaction was stopped by adding 100 μL of solubilization solution, then, formazan, formed by the cleavage of the yellow tetrazolium salt MTT, was measured spectrophotometrically by absorbance change at 550–600 nm using a microplate reader (BioRad (Hercules, CA, USA)). Six replicate wells were used for each group. The controls included untreated cells, whereas the medium alone was used as a blank.

### 4.6. Statistical Analysis

The data are represented as the mean ± standard error of the mean. *t*-tests and one-way analysis of variance were used to compare differences among groups, and statistical significance was assessed by the Tukey–Kramer post hoc test. The level of significance for all the statistical tests was set at *p* ≤ 0.05. All the statistics were run using the Prism 5.0a (GraphPad Software Inc., La Jolla, CA, USA) software package.

## 5. Conclusions

Taken together, our findings suggest that PKCε alteration could play a role in ALS pathophysiology, and PKCε agonism by Bryostatin-1 may represent a potential neuroprotective strategy against motor neuronal degeneration in a specific subgroup of sporadic ALS patients. The evidence reported here suggests that cellular-based in vitro models may be suitable to investigate specific molecular subgroups, thus representing an interesting starting point for future preclinical and clinical studies aimed at developing patient-tailored pharmacological treatments.

## Data Availability

Transcriptional data are available at EBI ArrayExpress database with the accession number E-MTAB-8635 (https://www.ebi.ac.uk/arrayexpress/experiments/E-MTAB-8635/ accessed on 1 June 2021) and at the Gene Expression Omnibus with the accession number GSE124439 (https://www.ncbi.nlm.nih.gov/geo/query/acc.cgi?acc=GSE124439 accessed on 1 January 2023).
